# Cloning and characterization of *Trypanosoma brucei* IFT complex B core

**DOI:** 10.1186/2046-2530-1-S1-P38

**Published:** 2012-11-16

**Authors:** C Pleban, G Dong

**Affiliations:** 1 Max F. Perutz Laboratories, Austria

## 

The cilium, a protrusion from the cell surface, has recently attracted immense attention due to its importance in cell signaling and its association with a wide range of human disorders. On the structural level, the cilium consists of a ring of nine microtubule doublets along which the bidirectional movement of ciliary building blocks and turnover products takes place. This transport is termed intraflagellar transport (IFT) and is carried out by two distinct multi-subunit protein complexes, IFT complex A and B, which are responsible for retrograde and anterograde transport, respectively. IFT complex B consists of at least 14 different proteins, eight of which are known to form a salt-stable core complex. Our research focuses on the IFT complex B core. The eight IFT genes from *Trypanosoma brucei* have been cloned into 4 Duet vectors (Novagen) to enable a simultaneous co-expression of the core complex. Upon co-expression in *E. coli*, the complex has been purified with minor impurities using Zinc-NTA affinity and size-exclusion chromatography. Six of the eight proteins have been confirmed via mass spectrometry and Western blot. On size-exclusion chromatography, different oligomeric states of the complex can be observed, ranging from >8MDa to around 600 kDa. Preliminary data suggests a liposome-binding capability of the complex, but the responsible subunit(s) remains to be elucidated. Since crystallization of the purified complex has not been successful, future studies will focus on electron microscopy analysis of the recombinant IFT complex B core.

